# E-HIP: An Energy-Efficient OpenHIP-Based Security in Internet of Things Networks †

**DOI:** 10.3390/s19224921

**Published:** 2019-11-12

**Authors:** Peter Kaňuch, Dominik Macko

**Affiliations:** Faculty of Informatics and Information Technologies, Slovak University of Technology in Bratislava, Ilkovičova 2, 842 16 Bratislava, Slovakia; xkanuch@stuba.sk

**Keywords:** energy efficiency, internet of things, low power, communication security, wireless sensor networks

## Abstract

The rapidly growing segment of the Internet of Things (IoT) makes the security threats more prominent than ever. The research around communication security and cybersecurity in such networks is still a challenge, mainly due to the typically limited energy and computation resources of IoT devices. The strong security mechanisms require significant power and thus the energy wastage must be minimized. Optimized application-specific security protocols are commonly used to make the data transfer more efficient, while still offering a high level of security. The supported security features, such as confidentiality, integrity or authenticity, should not be affected by the optimization. Our work is focused on optimizing one of the existing security protocols for the use in the IoT area, namely the Host Identity Protocol (HIP). Based on the analysis of related works, we have identified multiple possibilities for optimization and combined some of them into the proposed E-HIP optimized protocol. For verification purpose, it has been implemented as a modification of the open-source OpenHIP library and applied on a communication between real hardware devices. The secured communication worked correctly. The resulting effect of the proposed optimization has been evaluated experimentally and it represents an increase in energy efficiency by about 20%. Compared to other HIP optimizations, the achieved results are similar; however, the proposed optimizations are unique and can be further combined with some of the existing ones to achieve even higher efficiency.

## 1. Introduction

An ever increasing number of interconnected devices, creating the Internet of Things (IoT) [1], raises big concerns, especially from the security point of view. These devices are used in all possible areas of human life, such as industry, smart cities or agriculture [2], and many of them are critical—meaning that the damage of devices, failure of communication or uncovering sensitive data can cost financial losses, production delays, a decrease of life standard or even a human life. Network attackers started to focus on this mass-production market in a rather high volume, as indicated by a number of security incidences in past years [3,4]. Therefore, the security must be a priority when dealing with the IoT and a mass production and security features, such as authenticity, integrity, confidentiality or policy, must be supported [5,6,7].

However, another crucial property of IoT domain is that the end devices are often limited on the energy side, since they are powered by batteries or or some alternative power sources (e.g., energy harvesting). To prolong the lifetime of such devices and keep their maintenance (e.g., battery exchange) at minimum, IoT sensors are low-power devices with limited resources and it is therefore challenging to offer strong security features in them. The protocols managing and ensuring the security must be optimized for usage in the energy and resource constrained sphere of IoT. There are many research works on optimization and adjustment of security protocols [8], such as References [9,10,11]. However, no solution is perfect and generally applicable and thus the standard protocol must be optimized for the specific purpose of the intended application to avoid wasting of the energy.

UDP (User Datagram Protocol) is less energy demanding transport protocol than TCP (Transmission Control Protocol), because it is much simpler and has a smaller protocol header. Therefore, some approaches are focused on creating clones of protocols standardly operating above TCP to operate above UDP. The DTLS (Datagram Transport Layer Security) [12] protocol belongs to such clones. There also exist further optimizations of DTLS itself. The eeDTLS (Energy-Efficient DTLS) [9] protocol represents a DTLS optimization by reducing its message headers and modifying the handshake process. The Lithe [10] protocol represents a lightweight secure Constrained Application Protocol (CoAP) for IoT as a combination of DTLS and CoAP features to provide a secure data transmission and headers compression to improve energy efficiency. CoAP is probably the most widely used choice as an application protocol for IoT. However, it does not itself support security features to protect transferred data, such as authentication or encryption. Instead of integrating these features into the CoAP itself, such as the mentioned Lithe protocol, other protocols can be used to secure the communication (including the CoAP-based application layer), such as IPSec (Internet Protocol Security) [13]. It is quite resource intensive in its standard version; however, there exist some optimizations. For example, the LKA (Lightweight Key Agreement) [11] protocol represents a constrained version of the IKEv2 (Internet Key Exchange) protocol, offering minimal configurability (e.g., a single cryptographic algorithm to be used). As an alternative to IPSec/IKE, another key-exchange mechanism can be used, such as HIP (Host Identity Protocol) [14]. It was designed in such a way that enables to separate the device identification, represented by a cryptographic identifier, from its location, represented by the used IP address. It is a rare security feature that enables anonymous locations and supports mobility of devices. It is utilizable, even desired, in many IoT applications. Several research works were focused on HIP to optimize its energy requirements for easier use in IoT. The HIP-TEX (HIP Tiny EXchange) [15] protocol introduced a distributed computation of HIP to alleviate the limitations of IoT devices. The HIP-DEX (HIP Diet EXchange) [16] is another HIP modification that deals with computational limitations by using the less resource intensive Elliptic-curve cryptography. However, the HIP-TEX and HIP-DEX based solutions lack the perfect forward secrecy and anonymous locations security features of the original HIP. Even more security features are missing in the LHIP (Lightweight HIP) [17], although enabling its usage in the highly resource-conatrained mobile devices. An interesting analysis of the use of Elliptic-curve cryptography in HIP is provided in Reference [18]. The Slimfit [19] protocol further optimized HIP-DEX by compression of the HIP header, which effectively reduced the fragmentation rate. The compression approach was combined with the distribution approach in the CD-HIP (Compressed and Distributed HIP) protocol [20], which targeted 6LoWPAN (IPv6 over Low-Power Wireless Personal Area Networks) communication. Another distributed mechanism to alleviate the constrained devices is offered by CHIP (Collaborative HIP) [21], which uses a proxy-based key establishment.

This article, representing an extended version of our conference paper [22], is also targeted to HIP [14] optimization, since is offers interesting features for IoT applications (anonymous locations, mobility). We have analyzed the protocol and identified multiple possible optimizations to reduce its energy requirements. From the identified optimization possibilities, we have selected for implementation those not affecting the security level of the original protocol negatively. We have implemented the selected optimizations into an open-source OpenHIP library [23] and evaluated the results experimentally. This library was used because it is freely available; however, other protocol versions (even optimized) could be used for implementation (since the proposed modifications are unique), if their source code would be available. The key contributions of our work are as follows:

- identification of unique optimization possibilities (not used in existing works),
- increased energy efficiency of communication as well as computation (compared to the unmodified OpenHIP implementation),
- reduced bandwidth overhead and network load and
- minimized unnecessary waiting periods.

The article is organized according to the following structure. Section 2 outlines the background regarding the HIP processing and messaging. Section 3 contains the proposal regarding HIP optimization, in order to increase its energy efficiency. In Section 4, experimental evaluation of multiple proposed optimizations, implemented into the OpenHIP library, is described and discussed. The work is concluded in Section 5.

## 2. Background

**Figure 1:**
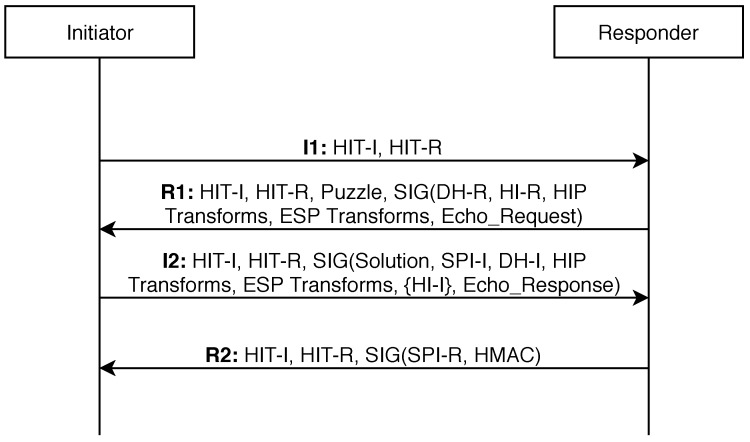
The Host Identity Protocol base exchange process.

This section contains basic information about the Host Identity Protocol (version 2) [14], which is designed to be used as a key-exchange mechanism to be used in cooperation with other protocols, such as ESP(Encapsulating Security Payload)/IPSec. In order to reduce transmitted data size, the HIP headers are used only while establishing the connection for parameters agreement. As previously mentioned, HIP is design to separate the device identification from the information about its location. Each HIP device has at least one host cryptographic identifier (it can have multiple identifiers), which enables to offer strong security features, such as authenticity, confidentiality, integrity and protection against various security threats (e.g., denial-of-service or man-in-the-middle attacks) [24]. These identifiers are not sent in the HIP messages but rather a 128-bit hash value is used, called Host Identity Tag (HIT). The protocol supports two kinds of devices: an initiator (e.g., an IoT end device) and a responder (e.g., an IoT gateway, a cloud server). The basic exchange of messages (called HIP-BEX) to establish a HIP association (an agreement on security parameters) consists of four key messages: I1 and I2 (the initiator is a sender), R1 and R2 (the responder is a sender). An overview of the HIP-BEX process (analyzed in [25]) is illustrated in Figure 1.

HIT-I and HIT-R represent host identity tags of the initiator and the responder, respectively. Puzzle represents a task to solve by the initiator (as a delay), which protects the responder against some denial-of-service attacks. Solution is the corresponding result of the puzzle, sent by the initiator. DH-I and DH-R represent the Diffie Hellman key exchange to create symmetric keys for data encryption. HI-I and HI-R represent host identifiers of the initiator and the responder, which correspond to the asymmetric public keys. HIP and ESP Transforms represent communication control parameters for the rest of the messages exchange. Echo_Request and Echo_Response represent test data to be exchanged. SPI (Security Parameter Index) identifies the HIP association (an instance). HMAC holds a control sum of the message. And finally, SIG is a digital signature.

**Figure 2:**
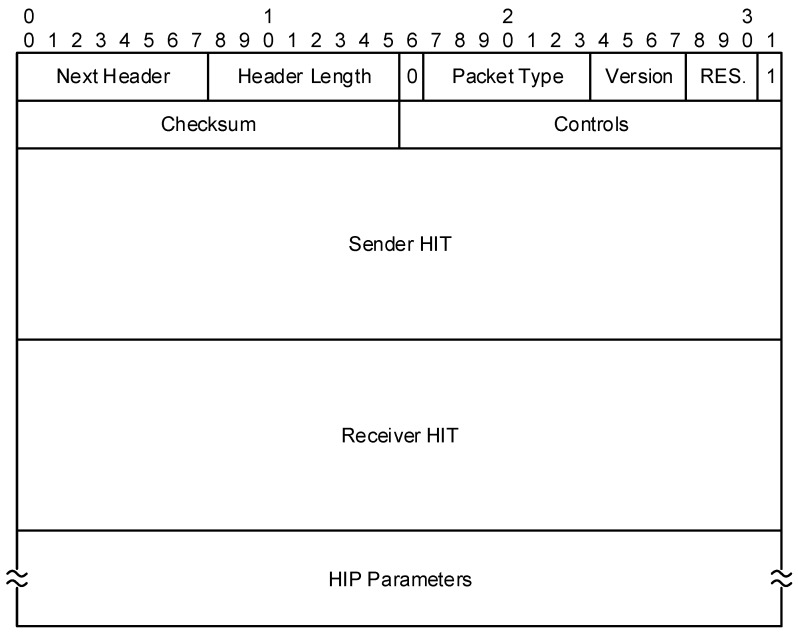
The HIP control messages header.

The format of HIP messages is illustrated in Figure 2. Besides the typical fields of common protocols, such as next protocol header information, a length of the protocol header, a version of the protocol, a type of the packet/message and control checksum, it contains also two 128-bit fields – for sender’s and receiver’s HITs.

**Figure 3:**
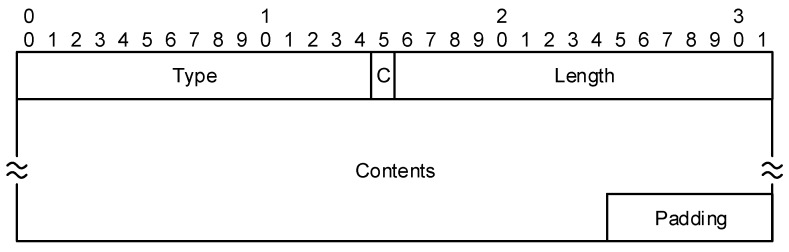
The HIP parameters header.

These two greatest header fields are followed by individual parameters in the wide-spread TLV (Type-Length-Value) form, illustrated in Figure 3. The numbered parameter types must be ordered in ascending manner. If such a condition is not followed, the message is assumed to be modified (i.e., tampered with) and the integrity is violated. A padding is used to adjust the message length to the standard 4-byte word size.

**Figure 4:**
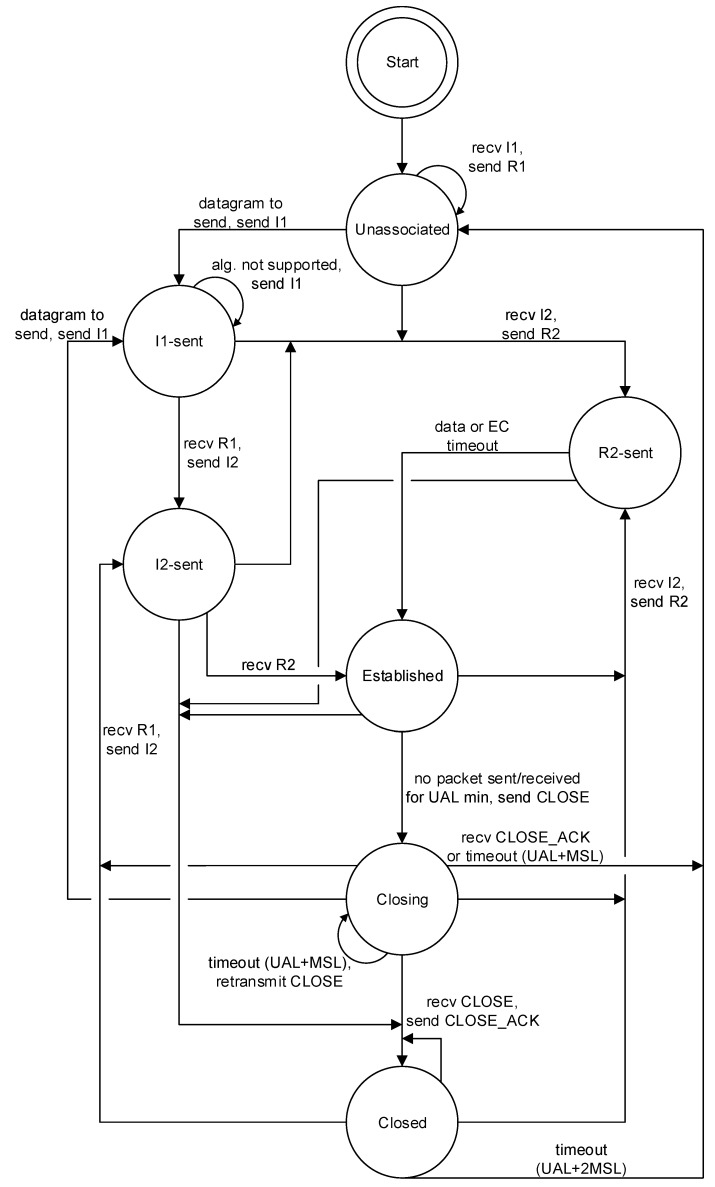
The state machine of the HIP protocol.

The HIP behavior is outlined by the illustrated state machine in Figure 4. It depicts the states and transitions between individual states at both ends of the connection. UAL (Unused Association Lifetime) represents a timeout of an active HIP association if no messages are sent or received. MSL (Maximum Segment Lifetime) is an expected maximum time, for which a HIP message is transmitted in the network. EC (Exchange Complete) is a timeout in the R2-sent, after which the device transits to the Established state even if no data are received yet. For example, based on the state machine, the common connection establishment is processed as follows. The initiator starts in the Unassociated state, from which it transits to I1-sent, then to I2-sent when R1 is received and to Established when R2 is received. The responder is in Unassociated until it successfully receives I2 and transits to R2-sent and to Established when data (an ESP packet) are received.

## 3. The Proposed Optimizations of HIP Energy Efficiency

An optimization of the standard protocols for the specific area of Internet of Things is quite common, as evidenced by the analysis of related works. The energy-constrained and performance-limited IoT devices just do not need full complexity and flexibility (i.e., configurability) of some standard protocols. For example, if the protocol requires an agreement on the algorithm to be used for data encryption but one of the communicating devices supports only a single algorithm, it will be always selected and thus the configuration-parameters negotiation is pointless. From the point of constrained IoT devices, unnecessary negotiations waste the precious energy.

In our work, we have targeted the HIP protocol, mainly because of its tempting features enabling anonymous location and mobility, useful for some IoT application, such as in healthcare or military sphere. We have stated the following requirements for our solution:

1. reduction of energy requirements of IoT end devices,
2. no negative impact on security,
3. comparison to other works,
4. correct function of the modified protocol,
5. easy application into IoT devices and
6. support of IPv4 or IPv6 communication.

We have identified several possible optimizations, targeting control plane of the communication (no data-plane impact):

- removal of the CLOSE_ACK message and the Closing state,
- parameters format reduction and
- the HI-R parameter removal.

The identified HIP modifications, combined into the proposed E-HIP solution, are more-closely described in the following subsections.

### 3.1. Removal of the CLOSE_ACK Message and the Closing State

Two HIP messages are used for termination of an HIP association, as can be deduced from the HIP state machine in Figure 4, namely the CLOSE message (initiating the termination procedure) and the CLOSE_ACK message (confirming the termination). A sending of the CLOSE message transits the device from the Established state to the Closing state, in which it waits for UAL+MSL time to receive the CLOSE_ACK message. If the confirmation message is lost (i.e., not received in time), the device wastes energy by waiting for a timer expiration (i.e., during this waiting period, it cannot be switched to an energy-saving state).

**Figure 5:**
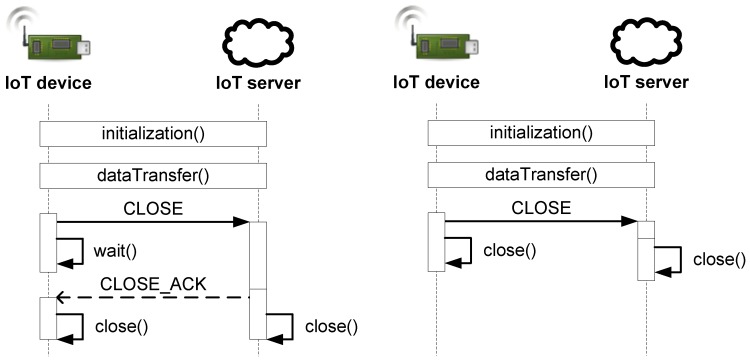
The termination process of the HIP association before (left) and after (right) the modification.

Therefore, we have proposed not to use the confirmation message. It has enabled us to eliminate the Closing state from the state machine and reduce the waiting period. It also saves the computing resources of the devices and some bandwidth of the communication channel, since the CLOSE_ACK message is not processed and transmitted. The device sends the CLOSE message and immediately terminates the connection (i.e., the association is not valid anymore). Upon receiving of the CLOSE message, the receiving devices also immediately terminates the connection (see Figure 5). If the CLOSE message is lost in the transmission, the backup timer is used to terminate the connection (no retransmission is used—that is, further energy is saved). Since it is not feasible for IoT end device to wait for a timer expiration, this solution is suitable for applications in which only an IoT device (i.e., initiator) can terminate the connection. The server (i.e., responder) is expected to be powered by the grid; thus, it is not problem for it to wait for a while if the CLOSE message is lost. This limits the general-purpose usage of the modified protocol; however, we prefer the increased energy efficiency. The IoT end device ignores the CLOSE message; thus, an attacker can not terminate the connection by repeating the sniffed server’s message. Also, since the CLOSE message includes a signature, the attacker is not able to close the connection on the server.

To implement this optimization into the OpenHIP library, it is necessary to modify the code in the *hip_handle_close()* function in the *src/protocol/hip_input.c* file:

1. sending and checking of the CLOSE_ACK message is deleted,
2. the Closed state setting is removed,
3. the function call of *delete_associations* is moved below setting of the Unassociated state.

### 3.2. Parameters Format Reduction

As mentioned in Section 2, four messages (I1, R1, I2 and R2) are exchanged during the HIP initiation phase (establishing association). In the R1 and I2 messages, several parameters are transferred (see Figure 1), each containing type and length information, as illustrated in Figure 3. The IoT communication is often predictable, especially when used for a periodic collection of sensor-measured data. The type and length of parameters can be preset. However, to transfer multiple parameters in a single message, a fixed order of parameters must be defined. In such a case, the type and length fields of parameters headers are no longer required in the messages and can be removed. This way, 16 bits are saved in each transmitted parameter. In the tested prototype, we have successfully reduced the I2 message size by 16 B.

**Figure 6:**
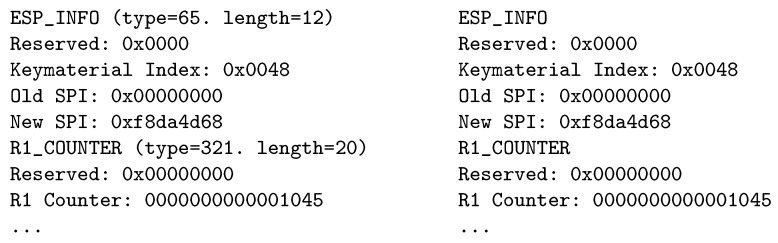
Packet contents before (left) and after (right) the modification.

Differences in the parameters messages contents before and after the proposed modification are illustrated in Figure 6.

An implementation of this optimization requires a removal of the redundant fields from the OpenHIP data structures, located in the *src/include/hip/hip_types.h* file. The creation and sending of the message must also be modified. The messages to be send are located in *src/protocol/hip_output.c*. Specifically for modification of the I2 message, the *hip_send_I2()* function has been changed: the original structures have been replaced by the modified structures (also in the called functions), the code parts using the removed parts of the structures have also been removed and positions of individual structures in the created messages have been ordered. Also, the receiving of the I2 message in the *hip_parse_I2()* function of the *src/protocol/hip_input.c* file.

### 3.3. The HI-R Parameter Removal

As described in Section 2, HI-R refers to the host identity of a responder (i.e., an IoT server), which represents its public key for asymmetric cryptographic algorithms, at least RSA (Rivest Shamir Adleman) and ECDSA (Elliptic Curve Digital Signature Algorithm) must be supported in HIPv2 [14]. Removal of this parameter also reduces the message size but also the computation resources for its processing. HI-R encodes the RSA key by a concatenation of information about the exponent length, the exponent and the modulus itself. The security level corresponds to the modulus length, which can be deduced from the previously mentioned explicit fields. The NIST organization [26] recommends to use the keys with the modulus length of at least 2048 b. Smaller keys must be changed more often to ensure they are not compromised. If an ECDSA key is used, it is encoded in HI-R by a concatenation of information about the curve label and the public key. The key size is in case of the ECDSA much shorter—that is, 160 b (the security level of the 1024 b RSA key) or 224 b (the security level of the 2048b̃ RSA key). If the public key is not transmitted as the HI-R parameter, it must be known to the initiator (i.e., an IoT end device) in some other way. Various IoT applications have various specific properties and requirements. In general, we have proposed two ways of public key distribution using the modified protocol, manual and automated.

#### 3.3.1. Manual Key Distribution

This solution involves a distribution of the server’s public key to the end device by uploading it to its memory manually, since it is not transmitted in the message exchange. This way, the length of the R1 message can be reduced by 128 B, that is, by 20% of the total message size (in case of the RSA keys). Because the key-pair must be changed after some period to ensure the security, a periodic regeneration of the server’s key imply it must be periodically distributed to all end devices. Also, the proposed manual distribution is beneficial from the security point of view, because it is more difficult for an attacker to find out the public key and to establish the connection. Since a manual distribution of the key is used, the system administrator must have an easy access to the end devices. Depending on the number of devices and their location, it might be quite challenging. Therefore, it is suitable for IoT applications in which all end devices are gathered in one place once a time, for example, for recharging purposes. An example of such applications can be the monitoring devices of patients’ health during clinical examinations at hospitals, which are recharged during a night or a weekend.

#### 3.3.2. Automated Key Distribution

**Figure 7:**
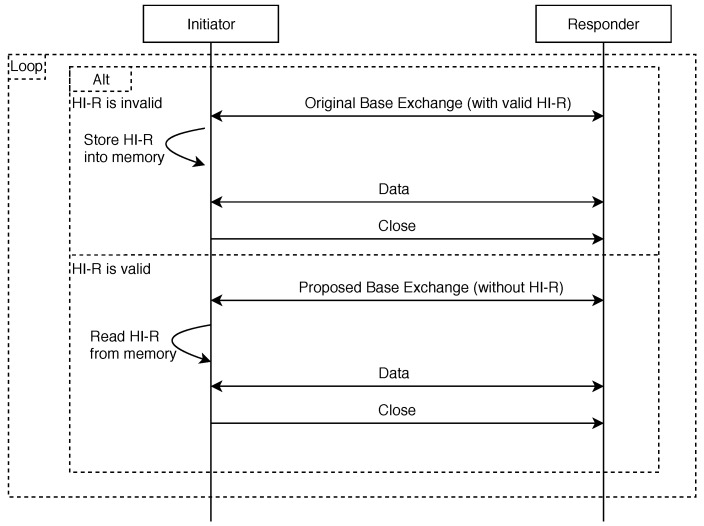
The proposed automated key distribution process.

Although the manual distribution is energy efficient (regarding the key transmission), it just cannot be used in some applications, where the end devices are distributed around a city. It would be too costly for an administrator to drive around and upload the keys (even if done only once a month). Therefore, an automated distribution is also proposed (illustrated in Figure 7), which does not require any administrator action.

The point is that the key is transmitted in the HIP message during initial communication; however, it is stored into the end device memory and is considered valid even after the HIP session is over (the association is terminated). For example, the restart of the end device or reinitialization of the protocol do not invoke a new key transmission. After the security timer expires (depending on the key modulus length), the key is regenerated and redistributed automatically. This solution is suitable for IoT applications with many end devices spread around huge area. An example of such application can be the monitoring devices of the available container capacity, distributed around the city.

An implementation of the manual key distribution involves a removal of the *build_tlv_hostid()* function call, which integrates it into a final message. It is also necessary to modify the processing of the received R1 message in the *hip_parse_R1()* function of the *src/protocol/hip_input.c* file. In this function, the parsing of a host identity form the message is replaced by loading from the *known_hi_filename* file. This file is generated by the server device using the modified functionality in the *src/util/hitgen.c* file to simplify its sharing.

### 3.4. Other Considered Optimizations

We have also considered other optimization possibilities described in Reference [22], which have been however found unsuitable for implementation. For example, since the sender and receiver HIT parameters take a significant portion of the HIP headers (as already shown in Figure 2), we have considered not using it and thus reduce the length of the exchanged messages even more. It could reduce 37% of the I1 message, 5% of the R1 message, 4.5% of the I2 message and 12.2% of the R2 message. However, the HIT parameter is not used just for identification purpose, it is also used in various security functions, such as validation of the puzzle solution or signature verification, and its removal could cause multi-homing problems and difficult interaction with middle-boxes (e.g., a firewall, a rendezvous server) [27]. Since one of our stated requirements was no negative impact on security, we have not implemented this idea. Further analysis of a suitable replacement for security functions must be done. For example, in data messages, the HIT parameter is replaced by the SPI parameter. However, a detailed impact on interaction with middle-boxes must be also analyzed and a suitable solution found to use such a modification.

### 3.5. Security Analysis

We have proposed three main modifications. In the first one, the original CLOSE message is not modified. Thus, from the security point of view, the authenticity of the HIP message is, like in the original HIP [14], verified using HMAC and SIGNATURE and the CLOSE message is ignored when no corresponding HIP association is found. Since only an initiator (i.e., an IoT end device) can terminate the connection, there is no way to close the connection by an attacker.

As we have mentioned in Section 3.2, we defined a fixed order of the parameters and removed information about the type and length. In our opinion, it has no impact on the security of the protocol, because of all security fields have been preserved in the message.

**Figure 8:**
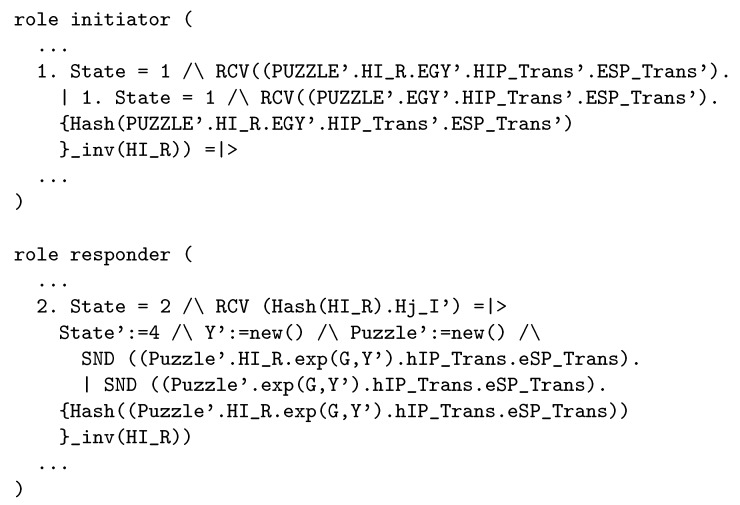
Modifications in the HLPSL code of the HIP protocol.

In the third proposed modification, we removed HI-R from the original message, which was a part of the four-way handshake. Due to making changes in the main part of the source code, it is necessary to verify security features. For formal verification, we decided to use the AVISPA software (http://www.avispa-project.org/), which is a software for the analysis of security-sensitive protocols. The high-level protocol specification language (HLPSL) is used for the formal description of the security protocol [28]. HIP has already been written in HLPSL language previously (http://www.avispa-project.org/library/hip.html). We have analyzed the existing HIP code in HLPSL in detail and modified it according to our proposal. The required changes are illustrated in Figure 8.

We have run the modified HIP protocol in the AVISPA software and we have obtained the same results as in the original protocol. (BACKEND OFMC -> SUMMARY safe, BACKEND OFMC -> GOAL As specified, BACKEND Cl-AtSe -> SUMMARY safe, BACKEND Cl-AtSe -> GOAL As specified).

## 4. Experimental Results

For verification whether the proposed E-HIP is functioning correctly, we have implemented a prototype in a form of a communication library. It is a modification of an open-source implementation of the HIP protocol, named OpenHIP (Source code: https://github.com/rektide/openhip, version: openhip-0.9) [23]. In the modified library, three proposed modifications were implemented, specifically the removal of the CLOSE_ACK message and the Closing state, the reduction of the parameters format and the removal of the HI-R parameter (the version of manual key distribution).

**Figure 9:**
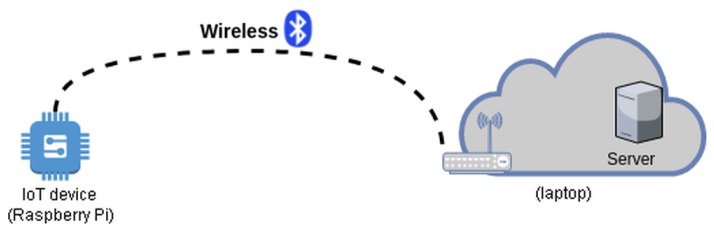
The experimental setup.

The experimental setup consisted of the Raspberry Pi 3 (RP) microcomputer with the Raspbian operating system, representing the IoT end device and the laptop computer, representing the cloud server. The topology is illustrated in Figure 9. Since the RP has the integrated Bluetooth 4.1 module, we have used Bluetooth as a communication technology, above which an IPv4 connection was established and the HIP protocol ensured a secure session for the data transfer.

To verify and evaluate the implemented optimizations, we have executed several experiments focused on various aspects.

**Figure 10:**
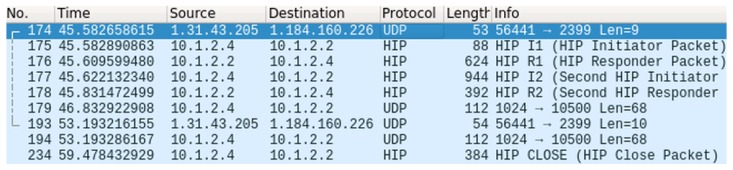
The optimized HIP communication, captured by Wireshark.

In the first phase, we have verified the overall function of the optimized protocol E-HIP. This verification was done on the above-mentioned real topology, using real devices with the modified library for communication. The Wireshark sniffer was used to observe the transmission of HIP messages, whether they are correct and received in the correct order. The captured communication is illustrated in Figure 10. The captured messages are briefly explained in Table 1.

The observed communication confirmed that the connection was successfully established, some data were transmitted and the connection was afterwards closed. Thus, we can conclude that the E-HIP functions correctly.

As a next experiment, the proposed E-HIP was compared to the original OpenHIP protocol. In the comparison, the key of 1024-bit was used. We have observed the number of transmitted (TX) and received (RX) messages and bytes, during the connection establishment and connection closing. Only two measurements were performed, since the same number of messages is always used; thus, a higher number of measurements would not provide different results. The results are provided in Table 2.

Based on the achieved results, we can see that communication efficiency of E-HIP is by approximately 20% higher than in case of OpenHIP. It indicates that the overall energy, required for transfer of some number of bytes, is reduced by the proposed optimizations.

In order to see how the E-HIP performs in case of 2048-bit key usage, we have compared the number of bytes transferred by individual HIP messages, especially by the R1 message during the connection-establishment phase. The contents of the original messages (OpenHIP) are illustrated in Figure 1 and described in Reference [14], while the modifications (E-HIP) are presented in Section 3. The results of such a comparison are provided in Table 3.

As we can see, the difference between E-HIP and OpenHIP in the number of transferred bytes is even higher in case of 2048-bit key (−22.25%). When we focus on the R1 message, we can observe the difference of −25.3% in case of 1024-bit key and −32.17% in case of 2048-bit key. Based on a computation (i.e., we have not measured that in communication between real devices), we expect that the difference in case of 4096-bit key would be approximately −38%. The reason is quite obvious, since the key is not directly transmitted in HIP messages in case of E-HIP.

The usage of 2048-bit RSA key increases the communication security; however, it obviously increases also the energy requirements. If we focus on the number of transferred bytes, it is increased by 40.14% when using OpenHIP (2048-bit) instead of OpenHIP (1024-bit). However, the usage of E-HIP (2048-bit) instead of OpenHIP (1024-bit) increases the number of transferred bytes only by 8.96%. Thus, the E-HIP can increase the communication security with a smaller increase of the energy requirements.

The next experiment was focused on evaluation of benefits of removing the CLOSE_ACK message and the Closing state from the protocol. Specifically, the time was measured that could be saved by not waiting for the acknowledgement message. The measurement was performed using the original OpenHIP protocol, in which the time between sending the CLOSE message and receiving the CLOSE_ACK message was targeted. We have executed the measurement 10 times and the average result was 84.35 ms. This time thus corresponds to the period, how sooner the devices can be switched to a power-saving sleep mode. It might seem a quite small amount of time; however, the testing was performed on the devices, the distance between which was approximately one meter. When considering the communication between a local IoT end device and a remote cloud-based server, the time would be much longer (due to a communication delay). The CLOSE_ACK message may also be lost and the IoT end device must wait even longer time for the timer expiration. This cannot happen in the proposed solution; thus, the energy of the energy-constrained IoT device is not wasted.

To evaluate the proposed optimisations in terms of power efficiency, we have measured the current consumption during HIP communication (using a multimeter, which was connected directly between the power supply and the RP device). The measurements were repeated 20 times and the average results are reported in Table 4. The test scenario was simple connection establishment and connection termination using E-HIP and OpenHIP protocols and observation of difference in the measured values. The power consumption is calculated using the formula of P=I×V, where *P* is the power, *I* is the current and *V* is the supply voltage. The idle operation represents the measured values, when the device was not communicating. The HIP power is thus deduced as a difference between the computed power consumption in some HIP operation and the idle power consumption.

Due to a very short period of time and the multimeter accuracy, we were not able to observe a difference for the connection termination when E-HIP was used. However, based on the results for connection establishment, we can see the increase in power efficiency by 20% when E-HIP is used instead of OpenHIP.

It is not easy to determine individual contributions of the proposed modifications to the total energy savings. However, we can estimate these contributions by using the numbers of spared bytes in the transmitted messages. The parameters format reduction contributed by approximately 1%, not sending CLOSE_ACK contributed approximately by 11.5% and the remaining approximately 7.5% is contribution of the HI-R parameter removal. In addition, more energy would be spared if the CLOSE_ACK message would be lost during transmission, since in our proposal the device does not need to wait for CLOSE_ACK to be received.

The implemented modifications have been carefully selected to not influence HIP messages parameters/fields that are used in computations of security algorithms. However, to be sure, the next experiment was focused on verification whether the modifications have not impacted the security characteristics (i.e., confidentiality, integrity, authenticity) of the HIP protocol. To provide these features, the HIP Base Exchange protocol supports various mechanism, such as puzzle computation, Diffie-Hellman key exchange or digital signature. The verification was performed for control messages during the connection-establishment phase. It was done by comparison of security notifications for individual operations of the E-HIP proposal and the original OpenHIP version. The important notifications that were monitored include:

- R1: the computed SHA1 values comparison,
- R1: RSA HIP signature is good,
- I2: the puzzle solution comparison,
- I2: HMAC verified OK,
- I2: RSA HIP signature is good,
- R2: HMAC_2 verified OK,
- R2: RSA HIP signature is good,
- HIP exchange complete,
- the created SPI comparison.

Checking these notifications during the protocol testing indicates that the proposed optimizations do not affect the security features of the original protocol. Therefore, the further security analysis is not required.

### Discussion

The experiments have proved the correct functioning of the proposed E-HIP protocol. It was tested above the widely spread IPv4 protocol; thus, it is usable in many applications requiring secure communication. It was also shown that E-HIP supports various key sizes, which makes it flexible and the benefits of the proposed modifications are even higher for longer (and thus more secure) keys. When we compare the improvements achieved by measurements of the number of transferred bytes and the measurements of the current consumption, we can see that both indicate the same increase in energy efficiency by 20%.

**Figure 11:**
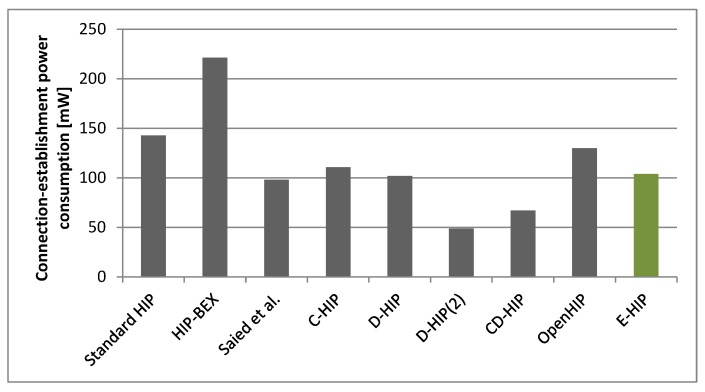
The comparison of various HIP versions power requirements.

In the experiments, we have shown that the proposed modification of the OpenHIP protocol is more power efficient to its original version. However, there exist other versions or modifications of HIP protocol. The comparison of various HIP versions is illustrated in Figure 11 (the power values are deduced from the similar existing works [15,20]).

As we can see, the achieved E-HIP power efficiency is comparable to some other modifications of the HIP protocol. The modifications based on distributed computation significantly outperforms other modifications (including ours); however, it is not quite suitable for our needs. We expect that the energy-constrained nodes would not waste their energy on computing tasks for other nodes. They are switched to power-saving mode when their computational power is not required. The proposed modifications are unique compared to others and thus they can be combined to increase the energy efficiency even more. The first two proposed modifications would not be affected by the used protocol version, since all of them use Closing state in the state machine and the acknowledgement. The third modification would bring smaller benefits when more efficient cryptographic algorithm, such as ECDSA, would be used, since it provides smaller keys offering the same security level. However, there still would be an increase in energy efficiency using the proposed optimizations. When used in combination with the compression-based optimizations, the proposed modifications could reduce compression/decompression processing, since they effectively reduce the message size. An investigation and evaluation of combined optimizations represents possible further work in this area.

It should be also noted that the proposed E-HIP protocol is not compatible with the standard one (i.e., it would not be compatible with other HIP devices, not using E-HIP version), due to the implemented modifications. However, an application-specific IoT communication requires application-specific protocols. If there would be need to preserve the compatibility, two versions could be implemented on the device (E-HIP and standard) and the standard (more energy intensive) version would be used only when required. It however depends on whether there is enough memory (to store both versions) in the constrained IoT device.

## 5. Conclusions

Although the optimization of security protocols for usage in the resource-constrained area of the Internet of Things is no new research direction, the pressure on energy efficiency is ever growing. There is still a need to extend lifetime of battery-operated devices or to integrate more functions into an energy-constrained device. In our work, we have targeted the OpenHIP open-source implementation of the HIP protocol. We have identified several optimization possibilities, from which we have selected those that do not influence HIP security characteristics. By the implementation of the selected optimizations into OpenHIP library, we have created E-HIP version of the HIP protocol, which is optimized for efficient use in the IoT sphere.

The experimental results confirmed increase in energy efficiency by approximately 20% compared to the original OpenHIP implementation. Specifically, it brings benefits regarding reduction of network load and protocol control overhead, reduced computation requirements or less energy-intensive securing of the communication. The unique E-HIP optimizations make it suitable for a combination with other optimizations of the related works, such as C-HIP, which opens a door for further energy improvements.

## Figures and Tables

**Table 1:** Description of captured HIP messages.

No.	Description
174	Data transfer over the HIP protocol.
175–178	Initialization and connection establishment.
179	Transmission of encrypted data over Bluetooth.
193	Data retransmission over the HIP protocol.
194	Encrypted data retransmission over Bluetooth.
234	Connection termination.

**Table 2:** Comparison of E-HIP and OpenHIP.

	OpenHIP	E-HIP	Difference
RX bytes	1184	760	−35.81%
RX messages	3	2	−33.33%
TX bytes	1048	1032	−1.53%
TX messages	3	3	0%
All bytes	2232	1792	−19.71%
All messages	6	5	−16.67%

**Table 3:** The number of transferred bytes in HIP messages of E-HIP and OpenHIP for different key size.

Message	OpenHIP (1024-bit)	OpenHIP (2048-bit)	E-HIP (1024-bit)	E-HIP (2048-bit)
I1	88	88	88	88
R1	664	920	496	624
I2	704	960	688	944
R2	264	392	264	392
CLOSE	256	384	256	384
CLOSE_ACK	256	384	0	0
Total	2232	3128	1792	2432

**Table 4:** Power measurements (measured voltage of 5.2 V).

Operation	Measured Current	Computed Power	Deduced HIP Power
Idle	310 mA	1.612 W	-
OpenHIP Establishment	335 mA	1.742 W	130 mW
E-HIP Establishment	330 mA	1.716 W	104 mW
OpenHIP Termination	320 mA	1.664 W	52 mW
E-HIP Termination	310 mA	1.612 W	0 mW

## Abbreviations

The following abbreviations are used in this manuscript:

6LoWPAN	IPv6 over Low-PowerWireless Personal Area Networks
CD-HIP	Compressed and Distributed HIP
CHIP	Collaborative HIP
CoAP	Constrained Application Protocol
DTLS	Datagram Transport Layer Security
EC	exchange complete
ECDSA	Elliptic Curve Digital Signature Algorithm
eeDTLS	energy-efficient DTLS
ESP	Encapsulating Security Payload
HIP	Host Identity Protocol
HIP-DEX	HIP Diet EXchange
HIP-TEX	HIP Tiny EXchange
HIT	Host Identity Tag
IKEv2	Internet Key Exchange version 2
IoT	Internet of Things
IPSec	Internet Protocol Security
LHIP	Lightweight HIP
lLKA	Lightweight Key Agreement
MSL	maximum segment lifetime
RP	Raspberry Pi 3
RSA	Rivest Shamir Adleman
SPI	Security Parameter Index
TCP	Transmission Control Protocol
TLV	type-length-value
UAL	unused association lifetime
UDP	User Datagram Protocol
